# Cardiac manifestations in pediatric COVID-19

**DOI:** 10.6061/clinics/2021/e3001

**Published:** 2021-07-26

**Authors:** Ana Carolina Marques do Vale Capucho, Paola Laureza Silva Resende, Daniel Alves Mascarenhas, Camila Lino Martins Rodrigues da Silva, Karen Saori Shiraishi Sawamura, Carolina da Rocha Brito Menezes, Maria de Fátima Rodrigues Diniz, Alessandro Cavalcanti Lianza, Werther Brunow de Carvalho, Clovis Artur Almeida da Silva, Gabriela Nunes Leal

**Affiliations:** I Unidade de Pediatria, Instituto da Crianca e do Adolescente (ICr), Hospital das Clinicas HCFMUSP, Faculdade de Medicina, Universidade de Sao Paulo, Sao Paulo, SP, BR.; II Laboratorio de Ecocardiografia, Instituto da Crianca e do Adolescente (ICr), Hospital das Clinicas HCFMUSP, Faculdade de Medicina, Universidade de Sao Paulo, Sao Paulo, SP, BR.; III Faculdade de Medicina FMUSP, Universidade de Sao Paulo, Sao Paulo, SP, BR.

## Cardiovascular manifestations in pediatric COVID-19

Since its very first description in Wuhan, Hubei province (China) in December 2019, the infectious disease of Coronavirus (COVID-19) has spread rapidly and diffusely around the world. This disease is caused by the Coronavirus 2 virus of the Severe Acute Respiratory Syndrome (SARS-CoV-2) and given its rapid spread, with consequences on an international scale, a pandemic was declared by the World Health Organization (WHO) on March 11, 2020 (1).

COVID-19 is rare in pediatric patients, with an incidence of less than 1% in children and adolescents aged less than 10 years. However, it can become a serious disease in 2.5% of this population, especially in those aged under 5 years (2) as the hospital admission rate is up to 20% in this population (3). The main serious complication in children affected by the disease is multisystemic inflammatory syndrome (MIS-C). This syndrome had a cardiovascular involvement in 80-100% of cases, with mortalities occurring in 67% of patients (2,3). Although adults with COVID-19 and cardiac disorders have an undoubtedly higher mortality rate, mortality may also occur in children and adolescents (4).

The objective of this editorial is to highlight the cardiovascular involvement in children and adolescents with COVID-19 since this has the potential for morbidity and mortality in this age group.

## Pathogenesis of cardiovascular injury caused by SARS-CoV-2

The involvement of the cardiovascular system in this viral infection can be explained in a multifactorial manner. Cardiotoxicity, the primary mechanism of injury to cardiac tissue, results from the entry of the virus into cells, hypoxia resulting from this disease, and adverse events of drugs used in the treatment of COVID-19. This direct viral invasion, postulated to be caused by the binding of the virus to angiotensin-converting enzyme 2 (ACE2) receptors on the cardiac surface, is based on previous studies that found viral RNA in 35% of myocardial infarctions in people infected with another coronavirus (SARS-CoV). This finding was reinforced in a publication in 2005 (5). Another more recent study (6) corroborates this hypothesis, with post-mortem findings in cardiac tissue of spherical viral particles on electron microscopy and viral RNA, confirmed by SARS reverse transcriptase-polymerase chain reaction. Compared with adults, children and adolescents are less susceptible to severe acute SARS-CoV-2 infection, possibly related to changes in the ACE2 receptor and the virus’ manner of entry into the intracellular medium (7,8).

In addition, some studies have reinforced the importance of immune-mediated injury in causing damage to the cardiovascular system. This is secondary to the excessive release of cytokines or deregulation of T cells generated by the presence of the virus, resulting in microvascular damage and endothelial dysfunction (5,9-11). Animal studies have also shown that elevated cytokines and inflammatory mediators led to decreased cardiac contractility, possibly mediated by calcium-dependent pathways (12-14). Inflammatory cytokines can also lead to peripheral vasodilation and consequent perpetuation of hemodynamic instability (1,2,12,15-17). Immune-mediated cardiovascular injury causes the systemic symptoms characterized by MIS-C. Studies on this syndrome correlated the levels of cardiac injury biomarkers and inflammatory markers with the activation of Th1 lymphocytes, even in a scenario of low viral load. There have also been reports of complete reversal of cardiac injuries after treatment with immunomodulatory agents, including systemic corticosteroids and intravenous immunoglobulins (1,12,13,15,16,18-21).

It is also plausible that SARS-CoV-2 infection increases the risk of thromboembolic and ischemic events. Although the mechanism of coagulopathy is unclear, it is likely to be multifactorial. Previous studies have shown that infections with other coronaviruses also elevated other components of the fibrinolysis pathway and regulated the genes associated with the induction of a procoagulant state. Patients with previous congenital heart disease are most prone to coagulation changes (13,16,20,21). Disseminated intravascular coagulation also causes myocardial injury and has been characterized in post-mortem biopsies of patients with SARS (22).

## Clinical spectrum of cardiovascular involvement in COVID-19

Myocarditis is usually diagnosed clinically by electrocardiograms, echocardiography, and increased cardiac enzymes since troponin is also defined as a marker of cardiac injury in children and adolescents (16,23). Myocarditis and pericarditis have been found in up to 40% and 25% of patients, respectively. These findings are associated with more severe disease and an increased risk of worse outcomes (24). Pericardial effusion occurred in up to 32% of patients. Together with the myocardial dysfunction findings, these characterize the pancarditis associated with COVID-19 (25).

Myocardial involvement may also be related to the presence of arrhythmias. In COVID-19, hypoxia, neurohormonal or inflammatory stress, and metabolic disorders contribute to changes in the cardiac rhythm. Some of the current drug therapies used in this disease, such as hydroxychloroquine, can also induce arrhythmia, adversely affecting cardiac electrophysiology (12,15,16,20,21,23,26). The ventricular dysfunction generated usually occurs on the left side. Hemodynamic instability and fluid-refractory hypotension requiring vasoactive drugs occurred in up to 44-47% of cases. Inflammatory cytokines can also lead to peripheral vasodilation and consequent perpetuation of hemodynamic instability (1,2,12,15-17).

Patients with COVID-19 have an increased risk of developing venous thrombosis, reaching 25%, with the highest risk in those with increased D-dimer and inflammatory markers, decreased fibrinogen, and those with the severe acute respiratory syndrome. There is suspicion mainly in patients who develop refractory hypoxemia or asymmetric edema of the lower limbs (22,27). Coronary thrombosis, in addition to the one being characterized, may correspond to one of the pathophysiological mechanisms of cardiovascular complications (10). Because of the systemic inflammatory response and imbalance in the oxygen supply, there is also an increased risk of coronary ischemia (22).

## Imaging findings

Transthoracic echocardiography is the most commonly used test to detect cardiovascular changes in children and adolescents with COVID-19. In most reports, changes have been observed mainly in the left ventricle, with a reduction in the ejection fraction (EF). Other findings included mitral regurgitation, pericardial effusion, and median hypokinesia of the inferoseptal and inferior walls (2,7,9,10,20,22,28). Oberweis et al. (21) reported a 21% reduction in left ventricular EF on hospital admission, with full recovery on day 10 of illness after treatment. In another series of cases, coronary aneurysm was found in 14.5% of the patients, two of whom had a Z score >10, indicating a giant aneurysm. It was more prevalent in patients with shock (2,7,9,28). Because of its applicability, this test is the initial method of choice in patients with suspected MIS-C to check for ventricular dysfunction, coronary artery aneurysms, and pericarditis (29).

The point-of-care ultrasound is used in the diagnosis of cardiovascular involvement and treatment. Its benefits are similar to transthoracic echocardiography, and it is useful for making real-time decisions at the bedside, including in suspected cases of MIS-C (30).

Cardiac magnetic resonance imaging, utilized for functional and morphological studies, is also useful for the differential diagnosis of cardiovascular diseases, such as acute ischemia. It also has interesting function in prognostication as the use of late gadolinium enhancement to identify areas of necrosis or fibrosis, which increase cardiovascular risk. In patients with suspected or diagnosed COVID-19, it is recommended to perform a short protocol (10-15 min), including a minimum set of data on cardiac function, focal myocardial damage, and three-dimensional imaging to identify coronary aneurysms (31). Examination of children and adolescents with COVID-19 is indicated for those who were diagnosed with MIS-C and those with an EF <50% or persistently low EF during the acute phase (29).

Cardiac computed tomography can assist in the exclusion of intracardiac thrombus and endocarditis. It may be preferable to transesophageal echocardiography because of the risk of aerosolization (28). However, in the pediatric population, this test should be only performed in those with suspected aneurysm of the distal coronary arteries that are not well visualized on echocardiography. In addition, this test is important in excluding coronary artery disease in patients with chest pain (31) and elevated troponin and diagnosing pulmonary thromboembolism, upon clinical suspicion (27).

## Post-mortem histological findings

Dolhnikoff et al. (6) confirmed the direct effect of SARS-CoV-2 on cardiac tissue, stressing that this virus is a fundamental contributing factor for myocarditis and heart failure in patients with MIS-C. Post-mortem cardiac ultrasonography also showed hyperechogenic and diffusely thickened endocardium, thickened left myocardium, and slight pericardial effusion. Post-mortem computed tomography angiography did not reveal any signs of coronary artery alterations.

## Diagnosis and treatment of MIS-C

MIS-C, a febrile inflammatory disease with mucocutaneous and gastrointestinal manifestations, is associated with multiple organ dysfunction (30,32). Coronaviruses have been shown to have tropism for the cardiac muscle. Similarly, there is a cardiac involvement in 80% of patients with MIS-C (16,18,19). As a result of the multisystemic immunoinflammatory event, the levels of inflammatory and interleukin markers, cardiac biomarkers, and abnormal coagulation parameters are usually elevated in laboratory tests, evidencing cardiovascular injury.

The management of this pathology proposes the need to attenuate the systemic inflammatory response and to provide supportive treatment to reduce the incidence of complications (18). In the case of signs of shock, expansion with crystalloids should be performed with caution because of the risk of severe myocardial dysfunction. When administering vasoactive drugs, which occurred in 47-95% of patients, inotropes are the most appropriate for MIS-C (2,12,14,17,23,32). The first treatment option is intravenous immunoglobulin, with the time of use ranging from 1-2 days (2,14,17,23,32). Pulse therapy with methylprednisolone should be administered to patients who have shown resistance to treatment with intravenous immunoglobulin.

These two medications can be concomitantly used in severe cases (30). In patients who are refractory to these medications, anakinra (interleukin-1 antagonist) or tocilizumab (interleukin-6 receptor antagonist) (22,24,29) can be used. Patients with COVID-19 are at risk for venous and arterial thromboembolic events. Therefore, anticoagulants should be prioritized (3,6,15,16).

## Cardiac complications and follow-up

The sequelae of pediatric COVID-19 can affect various organs and systems, such as the cardiovascular system. In fact, cardiac alterations associated with MIS-C were present in 35-100% of patients (11), with emphasis on the development of a coronary aneurysm, myocarditis, heart failure, cardiogenic shock, and pericardial effusion. This reinforces the need for prospective and serial imaging examinations (10,24).

In fact, in patients with MIS-C, the echocardiogram should be repeated after 1-2 weeks and after 4-6 weeks from the diagnosis. In patients with cardiac abnormalities during the acute phase, this test should be performed every 6 months (29).

## CONCLUSIONS

The cardiovascular system is frequently involved in pediatric patients with COVID-19 and presents with a wide clinical spectrum and high morbidity rate. Future multicentric and longitudinal studies involving a significant number of children and adolescents with COVID-19 and MIS-C will be necessary to assess the impact on this system.
